# Metabolic Reprogramming in Lung Cancer: Hallmarks, Mechanisms, and Targeted Strategies to Overcome Immune Resistance

**DOI:** 10.1002/cam4.71317

**Published:** 2025-10-29

**Authors:** Yi Chen, Menglin Bai, Ming Liu, Zhenshan Zhang, Chenxue Jiang, Kejin Li, Yun Chen, Yaping Xu, Leilei Wu

**Affiliations:** ^1^ Cancer Research Institute The Affiliated Cancer Hospital of Xinjiang Medical University Urumqi China; ^2^ Department of Radiation Oncology Qilu Hospital of Shandong University Jinan Shandong China; ^3^ Department of Radiation Oncology, Shanghai Pulmonary Hospital, School of Medicine Tongji University Shanghai China; ^4^ Department of Thoracic Surgery, Shanghai Pulmonary Hospital, School of Medicine Tongji University Shanghai China; ^5^ Department of Oncology, Nanfang Hospital Southern Medical University Guangzhou China

**Keywords:** immunosuppression, immunotherapy, lung cancer, metabolic reprogramming, tumor microenvironment

## Abstract

**Background:**

Lung cancer remains the leading cause of cancer‐related mortality worldwide. Although immunotherapy has revolutionized lung cancer treatment, its efficacy is often hindered by primary and acquired immune resistance. Increasing evidence indicates that metabolic reprogramming plays a crucial role in tumor progression and the establishment of an immunosuppressive tumor microenvironment in both non‐small cell lung cancer and small cell lung cancer.

**Methods:**

This review comprehensively summarizes recent preclinical and clinical findings on the metabolic alterations associated with immune suppression in lung cancer. The major metabolic pathways—glycolysis, lipid metabolism, and amino acid metabolism—were systematically analyzed, focusing on their regulatory enzymes, signaling pathways, and interactions with immune responses.

**Results:**

Metabolic reprogramming profoundly influences tumor–immune interactions. Enhanced glycolysis, dysregulated lipid synthesis and oxidation, and altered amino acid metabolism collectively contribute to immune evasion by impairing T‐cell activation, promoting regulatory immune cell populations, and modulating cytokine and checkpoint molecule expression.

**Conclusion:**

Targeting tumor metabolic remodeling offers a promising strategy to overcome immune resistance and improve the therapeutic efficacy of immunotherapy in lung cancer. Continued efforts to elucidate the molecular mechanisms linking metabolism and immunity may pave the way for novel combination therapies with durable clinical benefits.

**Abbreviations**
AAs: amino acids
ACC1: acetyl‐CoA carboxylase
ACLY: ATP citrate lyase
ACSL1: acyl‐CoA synthetase long‐chain family member 1
ASS1: argininosuccinate synthase 1
ATP: adenosine triphosphate
CAR: chimeric antigen receptor
circRNA: circular RNA
CPT1A: carnitine palmitoyltransferase 1A
ctDNA: circulating tumor DNA
DCs: dendritic cells
FAO: fatty acid oxidation
FAs: fatty acids
FASN: fatty acid synthase
GLS: upregulation of glutaminase
GLUT1: glucose transporter 1
HIF‐1α: hypoxia‐inducible factor 1α
HK2: hexokinase 2
HMGCR: HMG‐CoA reductase
HSP: heat‐shock protein
ICI: immune checkpoint inhibitors
IDO1: indoleamine 2,3‐dioxygenase
IDO2: tryptophan 2,3‐dioxygenase
LDHA: lactate dehydrogenase A
lncRNA: long noncoding RNA
LPL: lipoprotein lipase
LUAD: lung adenocarcinoma
LUSC: lung squamous cell carcinoma
MDSCs: myeloid‐derived suppressor cells
miRNA: microRNA
mTOR: mechanistic Target of Rapamycin
ncRNAs: non‐coding RNAs
NK: natural killer
NRF2: nuclear factor erythroid 2–related factor
NSCLC: non‐small cell lung cancer
PDKs: pyruvate dehydrogenase kinases
PD‐L1: programmed death‐ligand 1
PHGDH: phosphoglycerate dehydrogenase
PKM2: pyruvate kinase muscle isozyme 2
PPARs: peroxisome proliferator‐activated receptors
PUFA: polyunsaturated fatty acid
SCD1: Stearoyl‐CoA desaturase‐1
SCLC: small cell lung cancer
SLC7A11: solute carrier family 7 member 11
SREBP: sterol regulatory element‐binding protein
TAMs: tumor‐associated macrophages
TIME: tumor immune microenvironment
TME: tumor microenvironment
Tregs: regulatory T cells

## 1 Introduction

Despite advances in diagnostic techniques and therapeutic strategies, lung cancer remains the leading cause of cancer incidence and mortality worldwide [1], with non‐small cell lung cancer (NSCLC) accounting for approximately 85% of all cases [2]. Traditional treatment modalities such as surgery, radiotherapy, and chemotherapy have provided limited long‐term benefits, primarily due to the heterogeneity of tumor cells [2, 3, 4]. In recent years, immunotherapy, particularly immune checkpoint inhibitors (ICI), together with molecular targeted therapies has revolutionized the treatment landscape of lung cancer, markedly improving prognosis and prolonging patient survival [2, 3, 4, 5, 6]. However, only 20%–30% of patients achieve durable responses, even when immunotherapy is combined with other treatments, due to mechanisms of primary, adaptive, or acquired resistance [4, 7]. Tumor‐extrinsic factors, including insufficient T cell infiltration, upregulation of inhibitory immune checkpoints, and a dynamically regulated tumor microenvironment (TME), play critical roles in mediating immune resistance and evasion. The TME is now recognized as a key regulator of tumor initiation, progression, metastasis, and therapeutic response [8]. The complex interplay between cancer cells and the surrounding stromal, immune, and endothelial components forms a dynamic ecosystem that profoundly influences disease development and treatment outcomes.

The tumor immune microenvironment (TIME), a crucial component of the TME, comprises immune cells and their mediators that regulate the balance between anti‐tumor immunity and immunosuppression, profoundly shaping tumor progression and response to immunotherapy [3, 8, 9]. The TIME is regulated by a complex network of cellular and molecular interactions, among which tumor metabolic reprogramming serves as a key driver shaping its composition and function [10]. In lung cancer, including both NSCLC and small cell lung cancer (SCLC), cancer cells exhibit distinct metabolic features such as enhanced aerobic glycolysis, elevated de novo fatty acid synthesis, abnormal amino acids (AAs) utilization with a reliance on glutamine, and dysregulated cholesterol metabolism [8, 10]. These alterations not only sustain tumor growth but also remodel the TIME by suppressing effector T cell function and promoting immunosuppressive populations through key metabolites like lactate and cholesterol. Moreover, metabolic signaling pathways including hypoxia‐inducible factor 1α (HIF‐1α), sterol regulatory element‐binding protein (SREBP), and mechanistic Target of Rapamycin (mTOR) further integrate metabolic and immune regulation [8, 10]. Therefore, metabolic reprogramming in lung cancer is a central mechanism of immune suppression and therapeutic resistance, highlighting the potential of metabolic‐immune combination strategies.

This review focuses on the interplay between the TIME and metabolic reprogramming in lung cancer. It first outlines the metabolic alterations in both tumor and immune cells, then explores the clinical potential of combining metabolic intervention with immunotherapy. Uncovering the metabolic–immune crosstalk within the TIME may deepen our understanding of lung cancer progression and inform novel therapeutic strategies.

## 2 Current Status and Challenges of Immunotherapy in Lung Cancer

Lung cancer treatment is undergoing a major shift toward precision medicine, driven by advances in molecular targeted therapy and immunotherapy [2, 4, 11]. Molecular profiling has enabled more personalized treatment, with patients carrying driver mutations like *EGFR*, *ALK*, or *ROS1* benefiting from targeted inhibitors. However, around half of advanced NSCLC patients lack targetable mutations, and most eventually develop resistance, highlighting the indispensable role of immunotherapy [4]. ICI has reshaped the treatment landscape for advanced NSCLC [2, 5] and extensive‐stage SCLC [6], with pivotal trials such as KEYNOTE‐024, CheckMate 227, and IMpower133 establishing their role as first‐line therapies [12]. Their use is also expanding into neoadjuvant, adjuvant, and locally advanced settings, supporting a broader integration of immunotherapy throughout the disease course [2, 3, 6, 12].

Most patients do not derive substantial clinical benefit from immunotherapy; this resistance stems from both intrinsic factors, such as impaired antigen presentation, and extrinsic factors, including a suppressive TIME. Additionally, immune‐related adverse events such as pneumonitis, colitis, and dermatitis present important concerns for patient safety [13]. Current research is focused on addressing the limitations of immunotherapy efficacy through several strategies: (1) combination therapies, such as ICI combined with chemotherapy [5], antiangiogenic agents [14], radiotherapy [3], or metabolic modulators [10]; (2) improved biomarker selection [15], including programmed death‐ligand (PD‐L1) expression, circulating tumor DNA (ctDNA) [16], and integrative multi‐omic profiles [5]; and (3) the development of novel immunotherapeutic approaches, such as chimeric antigen receptor (CAR)‐T cells and CAR‐NK cells, bispecific antibodies, tumor vaccines, and microbiome‐based interventions [4]. Notably, metabolic reprogramming has emerged as a critical driver of TIME remodeling and represents a promising target to improve immunotherapy outcomes [4, 8, 10]. Tumor cells exhibit altered metabolism, which not only fuels rapid cell proliferation but also impairs immune responses through metabolic byproducts [10, 17]. Furthermore, metabolic signaling pathways are closely linked to immune regulation, highlighting potential avenues for synergistic therapeutic interventions [18, 19].

In summary, immunotherapy has become a cornerstone of lung cancer systemic treatment and continues to expand across disease stages. However, further advances depend on breaking through the current therapeutic ceiling. Integrating tumor metabolic reprogramming with immunomodulation represents a promising strategy to overcome resistance and improve durable responses in a broader patient population.

## 3 Hallmarks of Metabolic Reprogramming in Lung Cancer

### 3.1 Glucose Metabolism

Cancer cells preferentially rely on aerobic glycolysis, commonly referred to as the Warburg effect, whereby glucose is converted into lactate even in the presence of sufficient oxygen [20]. This phenomenon is characterized by elevated glucose uptake, increased lactate production, and enhanced generation of biosynthetic precursors such as nucleotides, lipids, and AAs. Although less efficient in adenosine triphosphate (ATP) yield, this metabolic reprogramming enables rapid energy production and supports the biosynthetic demands of uncontrolled proliferation [17, 19]. Lung cancer, in particular, exhibits a glycolysis‐dominant metabolic phenotype, with marked tumor‐type specificity and tissue‐of‐origin dependency [6, 10, 21].

Enhanced glycolytic activity is a hallmark of NSCLC, though with considerable heterogeneity across different subtypes [8, 10]. Lung squamous cell carcinoma (LUSC) represents a glycolysis‐predominant subtype, characterized by high expression levels of glucose transporter 1 (GLUT1), hexokinase 2 (HK2), and lactate dehydrogenase A (LDHA), as well as strong FDG uptake on PET imaging [22, 23]. These features confer increased metabolic targetability; however, they are also associated with relatively rigid metabolic pathways, which may reduce the tumor's ability to adapt to environmental changes [10, 22]. In contrast, lung adenocarcinoma (LUAD) displays greater metabolic flexibility, capable of switching between glycolysis, fatty acid oxidation (FAO), glutamine metabolism, and oxidative phosphorylation, depending on genetic drivers and microenvironmental cues (e.g., hypoxia, nutrient availability) [17, 22]. Although heterogeneity exists, NSCLC generally exhibits greater metabolic plasticity compared to SCLC. Accordingly, PET‐based metabolic indicators serve as valuable prognostic markers in NSCLC [23]. SCLC demonstrates the highest glycolytic activity among lung cancer subtypes, driven by rapid proliferation and neuroendocrine‐associated transcription factors such as MYC and NEUROD1, which upregulate HK2, LDHA, and pyruvate kinase muscle isozyme 2 (PKM2) [21, 24].

Glycolysis, the primary metabolic pathway for energy production in tumors, plays a significant role in shaping the TME. This metabolic reprogramming leads to the production of lactate, which acidifies the TME, thereby promoting immune suppression. Lactate accumulation can inhibit the activation and function of effector T cells while enhancing the activity of immunosuppressive cells such as regulatory T cells (Tregs) [10]. Additionally, glycolytic intermediates like pyruvate and acetyl‐CoA contribute to the biosynthesis of lipids and AAs, further supporting tumor cell proliferation. The reliance on glycolysis also alters the immune cell landscape, as glycolytic inhibition has been shown to restore T cell function and enhance antitumor immunity [25].

### 3.2 Lipid Metabolism

Lipids, including glycerophospholipids, triglycerides, fatty acids (FAs), sphingolipids, and sterol lipids, are fundamental to membrane structure and energy metabolism [10, 17]. In both NSCLC and SCLC, lipid metabolism is reprogrammed in a subtype‐specific manner to support tumor growth, particularly under hypoxia, nutrient limitation, or treatment pressure [8, 22]. This reflects the metabolic adaptability of lung cancer. FAs originate from two main sources: exogenous uptake and endogenous (de novo) synthesis [18]. Tumor cells increasingly rely on de novo fatty acid synthesis, supported by key enzymes such as ATP citrate lyase (ACLY), acetyl‐CoA carboxylase (ACC1), fatty acid synthase (FASN), and stearoyl‐CoA desaturase 1 (SCD1), which together promote membrane biogenesis, signaling, and energy supply.

Aberrant cholesterol metabolism is another metabolic hallmark of NSCLC [17]. Tumor cells upregulate HMG‐CoA reductase (HMGCR) and SREBP pathways to boost cholesterol synthesis. Cholesterol accumulation in lipid rafts stabilizes membrane‐bound proteins such as EGFR and contributes to drug resistance. Notably, the cholesterol derivative 27‐hydroxycholesterol is enriched in NSCLC and exerts dual roles: activating liver X receptor signaling to suppress EGFR‐mutant cell proliferation, while also promoting bone metastasis and resistance via PI3K/STAT3 pathway activation [26].

In contrast, studies on lipid metabolism in SCLC are relatively limited. Nevertheless, increasing evidence suggests that SCLC exhibits a high dependency on fatty acid synthesis and membrane lipid remodeling, particularly within stem‐like or drug‐resistant subpopulations [6, 21, 24]. SCLC appears to lack the metabolic plasticity observed in NSCLC and may rely more rigidly on lipid biosynthetic pathways to sustain rapid proliferation and maintain its neuroendocrine phenotype.

Lipid metabolism, particularly fatty acid synthesis, is another key metabolic pathway that influences the TME. The accumulation of FAs and lipids not only fuels tumor cell proliferation but also remodels the immune landscape within the TME [27]. FAs like palmitate can be incorporated into membrane phospholipids, enhancing cell membrane integrity and promoting immune evasion [28]. Moreover, the shift in lipid metabolism affects immune cells, with FAs influencing the differentiation and function of macrophages, dendritic cells (DC), and T cells. For example, elevated lipid levels have been associated with macrophage polarization into an M2 phenotype, which promotes tumor progression and suppresses anti‐tumor immune responses [10]. Targeting FASN and other lipid metabolic pathways holds potential for reprogramming the immune environment to enhance the efficacy of immunotherapy. Transcription factors such as SREBP1 and peroxisome proliferator‐activated receptors (PPARs) enhance lipogenesis and contribute to the polarization of immunosuppressive tumor‐associated macrophages (TAMs) [29]. FAO facilitated by carnitine palmitoyltransferase 1A (CPT1A) enhances metabolic fitness in Tregs and myeloid‐derived suppressor cells (MDSCs), reinforcing immune evasion [30]. Activation enzymes like acyl‐CoA synthetase long‐chain family member 1 (ACSL1) and lipoprotein lipase (LPL) and polyunsaturated fatty acid (PUFA)‐metabolizing enzymes generate immunomodulatory lipids that inhibit CD8^+^ T cell function [31]. Collectively, altered lipid metabolism reshapes the TIME, offering targets for metabolic‐immunologic therapies.

### 3.3 Amino Acid Metabolism

Amino acid metabolic reprogramming in lung cancer encompasses multiple interconnected pathways, with glutamine, serine, glycine, tryptophan, and arginine being the most prominently affected [6, 21, 24]. Among these, glutamine metabolism is the most extensively studied and functionally significant. Although glutamine is considered a non‐essential amino acid under normal physiological conditions, it becomes conditionally essential in rapidly proliferating tumor cells. In addition to serving as a key carbon and nitrogen source for the synthesis of nucleotides, AAs, and FAs, glutamine also supports redox homeostasis through glutathione production. Lung cancer cells exhibit a marked dependence on glutamine, facilitated by upregulation of transporters such as alanine, serine, cysteine transporter 2 (ASCT2/SLC1A5) and enhanced glutaminase activity to drive uptake and catabolism [32]. In parallel, upregulation of solute carrier family 7 member 11 (SLC7A11) promotes cystine uptake for glutathione biosynthesis, and enhanced one‐carbon metabolism via phosphoglycerate dehydrogenase (PHGDH) fuels nucleotide synthesis and epigenetic modifications [33]. Although data are limited in SCLC, early evidence suggests that subsets of SCLC may also exhibit dependency on amino acid metabolism, potentially reflecting unique metabolic vulnerabilities amenable to therapeutic targeting [6, 24, 33].

Amino acid metabolism—particularly glutamine and arginine—also critically remodels the TME. For example, the upregulation of glutaminase (GLS) in tumor cells leads to the production of glutamate, which can suppress T cell activity and promote tumor immune evasion [34]. Furthermore, tryptophan metabolism is redirected toward the immunosuppressive kynurenine pathway via indoleamine 2,3‐dioxygenase 1 (IDO1) and tryptophan 2,3‐dioxygenase (TDO2), leading to suppression of CD8^+^ T cell and induction of immune tolerance [34]. Arginine metabolism is similarly dysregulated, with decreased expression of argininosuccinate synthase 1 (ASS1) and increased ARG1 activity, resulting in arginine depletion and T cell dysfunction [35]. Thus, targeting amino acid metabolism represents a promising therapeutic approach to reprogram the TME and enhance immune responses.

### 3.4 Other Forms of Metabolic Reprogramming in Lung Cancer

Beyond glycolysis, lipid, and amino acid metabolism, lung cancer undergoes additional metabolic reprogramming that underpins tumor progression and immune evasion [8, 21, 33]. Nucleotide metabolism is notably upregulated, with enhanced purine and pyrimidine biosynthesis fueling nucleic acid synthesis and contributing to therapeutic resistance. One‐carbon metabolism, tightly linked to serine/glycine utilization and the folate–methionine cycle, supports DNA methylation, redox balance, and anabolic growth [33]. Lung cancer displays widespread dysregulation of metal metabolism involving iron, copper, zinc, manganese, calcium, and magnesium, which collectively shape redox homeostasis, immune suppression, and resistance to cell death [36]. In addition, emerging evidence indicates that targeting the heat‐shock protein (HSP) network to therapeutically reprogram mitochondrial metabolism can overcome metabolic plasticity and therapy resistance in NSCLC [37]. Despite being largely in the early stages of investigation, these noncanonical metabolic pathways have attracted growing interest for their contributions to tumor progression and immune regulation. They not only support cancer cell growth and survival but also reshape the immune landscape, offering new opportunities for metabolic immune combination therapies.

## 4 Rewired Metabolism Shapes Immunosuppressive Niches in Lung Cancer

### 4.1 T Cells

T cells, much like tumor cells, rely heavily on glycolysis to sustain their activation, proliferation, and cytotoxic function [19]. In lung cancer, tumor cells exhibit markedly elevated glycolytic activity, leading to glucose depletion within the TME and directly impairing CD8^+^ T cell glycolysis and effector capacity [8, 17]. In parallel, metabolic reprogramming in tumor cells results in the accumulation of lactate and the establishment of local hypoxia. Excess lactate not only acidifies the TME but also inhibits lactate export from T cells, thereby disrupting their ability to maintain aerobic glycolysis and further reducing their functional fitness [38]. Moreover, effector T cells within the TME face severe metabolic constraints, including limited availability of AAs and accumulation of immunosuppressive metabolites such as kynurenine and cholesterol [10, 17, 18, 19]. These factors collectively impair mTOR signaling, suppress T cell receptor signaling, and drive T cell exhaustion. Together, these metabolic barriers orchestrated by tumor cells severely hinder T cell‐mediated antitumor immunity and contribute to immune escape in lung cancer.

In contrast to effector T cells that rely heavily on glycolysis for their antitumor function, Tregs exhibit a distinct metabolic profile adapted to the nutrient‐deprived and hypoxic TME [39]. Tregs preferentially utilize FAO and oxidative phosphorylation for energy production, enabling them to thrive under glucose‐limited conditions that impair effector T cell function. Moreover, tumor‐derived metabolites further enhance Treg proliferation and suppressive capacity, reinforcing their immunosuppressive role and contributing to immune evasion.

### 4.2 Macrophages

Macrophages represent the most abundant immune cell population within the TIME and play pivotal roles in orchestrating immune regulation and metabolic adaptation [18, 19]. In response to local signals, TAMs exhibit functional plasticity and can polarize into either pro‐inflammatory M1 or immunosuppressive M2 phenotypes. Notably, M2‐like TAMs are key mediators of tumor‐promoting immunosuppression. Their polarization is driven by tumor‐derived lactate, which stabilizes HIF‐1α and enhances arginase expression, thereby reprogramming macrophage metabolism to suppress effector T cell activity, promote immune evasion, and facilitate angiogenesis [40].

### 4.3 Dendritic Cells

DCs as professional antigen‐presenting cells are essential for initiating adaptive immune responses. However, tumor‐derived lactate impairs DC differentiation and antigen‐presenting capacity, thereby blunting T cell priming. Moreover, lipid accumulation within tumor‐associated DCs disrupts antigen processing via MHC class II pathways and reduces their ability to stimulate T cells, contributing to the establishment of an immunosuppressive milieu.

### 4.4 Natural Killer Cells

Natural killer (NK) cells play a crucial role in innate immune surveillance. However, their cytotoxic function is markedly impaired by the immunosuppressive metabolic environment. Tumor‐derived lactate has been shown to suppress NK cell activation and cytokine production, while MDSCs further inhibit NK cell responses, thereby weakening early antitumor defenses.

### 4.5 Myeloid‐Derived Suppressor Cells

MDSCs are key mediators of tumor‐induced immunosuppression and are often expanded in response to metabolic cues from the tumor. Lactate accumulation in the TIME promotes MDSC expansion and enhances their suppressive activity. MDSCs inhibit T cell function through multiple mechanisms, including the depletion of L‐arginine and the transfer of immunosuppressive metabolites such as methylglyoxal, further exacerbating immune evasion and tumor progression.

The key metabolic pathways and their effects on the function of immune cells in lung cancer are listed in Table 1 [10, 25, 27, 38, 41, 42, 43, 44, 45, 46, 47, 48, 49]. Recent studies have emphasized that, in addition to the three major metabolic pathways, specific metabolites and trace elements play crucial roles in modulating the TME. As previously discussed, metabolites such as lactate, FAs, and kynurenine not only serve as essential energy sources for tumor cells but also significantly influence immune cell function within the TME. Similarly, trace elements such as zinc and selenium have been implicated in modulating immune responses and influencing cancer progression. Zinc, which is critical for immune cell function, can regulate T cell differentiation and cytokine production [50], while selenium, known for its antioxidant properties, has demonstrated potential in inhibiting tumor growth through its effects on cellular stress responses [51].

**TABLE 1:** Key metabolic pathways and their effects on immune cell function in lung cancer.

Immune cell type	Cell subtypes	Glycolysis	Lipid	Amino acid	References
T cell	Naïve T cell	—	—	Depletion of glutamine blocks Naïve T cell proliferation and cytokine production	[38]
	Treg cell	High levels of lactate increase Treg cells	Lipid‐rich TME supports Treg cells' survival and function	Growing IDO1 activity facilitates Treg activation	[10, 41, 42]
	Effector T cell	Lactic acid suppressed the proliferation and cytokine production of cytotoxic T lymphocytes (CTLs) and led to a decrease in cytotoxic activity	—	Growing IDO1 activity prevents T effector activation	[38, 42, 43]
	CD8+ T cells	Glucose consumption induces senescence CD8+ T cells. High levels of lactate also inhibit the function of CD8+ T cell	Lipid overload leads to mitochondrial dysfunction, increased ROS, T cell exhaustion, and reduced cytotoxic activity; FAO can strengthen CD8^+^T cell responses during anti‐PD‐1 therapy	Loss of Glutaminase promotes differentiation and effector function of CD8 CTL cells	[10, 25, 41, 44]
	CD4+ T cells	Glucose consumption induces senescence of CD4+ T cells	Enhanced FAO supports Th1 activity, promotes antitumor immunity, contributes to immunosuppression (Th17)	Loss of Glutaminase promotes differentiation and effector function of CD4 Th1 cells	[41, 44]
	Memory T cell	—	Tissue‐resident memory T lose functionality when deprived of fatty acids; Suppressing FAO hinder the transition from effector to memory phenotype	—	[27, 45]
Macrophages	M1	—	Fatty acid accumulation leads to a suppression of M1 macrophages inflammatory responses and a reduced capacity to attack tumors	—	[41]
	M2	High levels of lactate polarize macrophages toward the M2 phenotypes	FAs can promote the accumulation of M2 macrophages	—	[10, 46]
NK cell	—	Lactate and acidification Inhibite NK cells' effector functions	—	Growing IDO1 activity dampens NK cell cytotoxicity and proliferation	[10, 42, 47]
Monocyte	—	High levels of lactate inhibite monocyte migration and activation, inhibite cytokine release of TNF and IL‐6	—		[48, 49]
Dcs	—	High levels of lactate prevent dendritic cell differentiation, inhibit antigen presentation	Lipids impair DCs' ability to process antigens	Growing IDO1 activity drives the expansion and activation of DCs	[10, 48, 49]
MDSC	—	Lactate accumulation promotes the expansion of MDSCs and enhances their suppressive activity	—	Growing IDO1 activity drives the expansion and activation of MDSCs	[10]

Abbreviations: Dc, dendritic cell; FAO, fatty acid oxidation; IDO1, indoleamine 2,3‐dioxygenase 1; IL, interleukin; MDSC, myeloid‐derived suppressor cell; NK, natural killer; ROS, reactive oxygen species; TME, tumor microenvironment; TNF, tumor necrosis factor.

In addition, preclinical studies investigating dietary interventions have shown promise in modulating the TME and enhancing therapeutic responses. Diets enriched with specific nutrients, such as omega‐3 FAs and polyphenols, have been reported to alter tumor metabolism and activate immune responses. For instance, omega‐3 FAs, commonly derived from fish oil, have been shown to reduce inflammation and inhibit tumor growth [52], while polyphenols from fruits and vegetables influence metabolic pathways that support immune cell function [53]. These findings suggest that dietary interventions could serve as a valuable adjunctive strategy to enhance the efficacy of conventional therapies and immunotherapy, potentially improving clinical outcomes in cancer treatment.

## 5 Targeting Metabolic Reprogramming to Enhance Immunotherapy in Lung Cancer

### 5.1 Targeting Glucose Metabolism

Aerobic glycolysis is a hallmark of metabolic reprogramming in lung cancer and functions as a dominant pathway that modulates multiple immune cell subsets within the TIME [10, 17]. This coordinated metabolic interplay not only fosters a profoundly immunosuppressive milieu but also gives rise to the concept of “metabolic immune checkpoints” [54], wherein tumor‐intrinsic metabolic rewiring directly hampers effective immune surveillance. Consequently, targeting glycolytic pathways represents a promising immunometabolic checkpoint strategy to improve therapeutic outcomes in lung cancer. Current approaches primarily focus on inhibiting key glycolytic enzymes such as HK2 [55], LDHA [40, 56], pyruvate dehydrogenase kinases (PDKs) [57], and GLUT1 [58] and targeting critical regulatory pathways including HIF‐1α and PI3K/Akt/mTOR that sustain the crosstalk between metabolism and immune suppression.

In lung cancer, aberrant activation of HIF‐1α is associated with poor prognosis, enhanced glycolytic activity, and an immunosuppressive TME [56]. Although direct inhibition of HIF‐1α remains challenging due to its nature as a transcription factor, alternative strategies targeting its upstream regulators such as PI3K/Akt/mTOR or downstream effectors including PDK1 and LDHA have shown promise in preclinical studies. The PI3K/Akt/mTOR pathway is a central driver of metabolic reprogramming in NSCLC, integrating extracellular signals to promote glucose uptake, glycolytic enzyme expression, and anabolic activity [56]. Its activation leads to lactate accumulation and TME acidification, fostering effector T cell exhaustion and Treg stability. Exogenous stimuli such as serotonin and PM2.5 can also engage this axis, amplifying glycolysis and immune suppression [59]. Everolimus, the only clinically approved mTOR‐class inhibitor, though not indicated for NSCLC, has shown potential in preclinical studies to overcome EGFR‐TKI resistance and enhance antitumor efficacy. These findings underscore the therapeutic value of targeting the PI3K/Akt/mTOR pathway, particularly in combination with ICI, to improve immunotherapy outcomes in NSCLC.

### 5.2 Targeting Lipid Metabolism

Targeting lipid metabolism in lung cancer is still in early exploration, with mechanistic studies underway and several agents in phase I/II trials. Inhibitors of FASN (e.g., TVB‐2640 [60]) and ACC (e.g., ND‐646) exert antitumor effects by impairing membrane biosynthesis, disrupting redox balance, and overcoming therapy resistance. Blocking FAO through CPT1A inhibition (e.g., etomoxir) shows promise in suppressing immunosuppressive cell subsets, though clinical use is limited by toxicity [61]. Statins, via inhibition of the mevalonate pathway, have been retrospectively associated with improved responses to immunotherapy [62]. Additionally, targeting cholesterol esterification (e.g., ACAT1 inhibitors) and lipid signaling pathways (e.g., COX‐2 inhibitors) has been shown to modulate immune cell function and reshape the TIME [19]. Collectively, lipid metabolism has emerged as a tractable target to overcome immune evasion and enhance antitumor efficacy, though clinical translation remains in its early stages.

### 5.3 Targeting Amino Acid Metabolism

In lung cancer, therapeutic strategies targeting amino acid metabolism focus on disrupting glutamine, arginine, and tryptophan pathways through enzyme inhibition or transporter blockade. Glutamine and tryptophan metabolism have been among the earliest amino acid pathways targeted in lung cancer. Telaglenastat, a selective glutaminase inhibitor, failed to improve outcomes when combined with Nivolumab in ICI‐resistant NSCLC [63], though its combination with an mTORC1/2 inhibitor showed modest disease control in advanced settings [64]. Similarly, despite early enthusiasm, IDO1 inhibitors targeting the tryptophan‐kynurenine‐AHR axis failed to demonstrate meaningful clinical benefit, either as monotherapy or in combination with ICI [40, 56]. These outcomes reflect the challenges of targeting immunometabolic suppression in resistant tumors.

In parallel, arginine and cysteine metabolism have emerged as promising therapeutic targets. ASS1‐deficient NSCLC exhibits arginine auxotrophy [65], and arginine deprivation with ADI‐PEG 20 has shown preliminary efficacy in combination with chemotherapy [66], though monotherapy yielded no responses in ASS1‐deficient SCLC. Cysteine metabolism regulates ferroptosis sensitivity through SLC7A11‐mediated cystine uptake, contributing to resistance in refractory tumors [56, 67]. Several cysteine‐targeting compounds have shown preclinical efficacy but remain in early development.

### 5.4 Ongoing or Recent Clinical Trials Targeting Tumor Metabolism in Lung Cancer

Metabolic reprogramming plays a pivotal role in tumor growth, metastasis, and therapeutic adaptation [68, 69]. Recent clinical trials have increasingly focused on targeting metabolic pathways as a strategy to enhance the treatment of lung cancer. For instance, PX‐478, a HIF‐1α inhibitor, has completed a Phase I trial, demonstrating limited efficacy but acceptable tolerability in NSCLC patients [70]. Similarly, Sapanisertib (TAK‐228), an mTORC1/2 inhibitor, has yielded modest results, with a partial response rate and a disease control rate (DCR) of 37% in NSCLC. In the domain of lipid metabolism, TVB‐2640 (Denifanstat), a FASN inhibitor, is currently undergoing a Phase II trial. This investigational agent aims to disrupt FASN, with ongoing evaluation to assess its impact on tumor progression and immune modulation within the TME. On the amino acid metabolism front, pegargiminase (ADI‐PEG20), an enzyme that depletes arginine, is being tested in combination with chemotherapy in clinical trials. Preliminary data indicate promising results, with a DCR of 85.7% in NSCLC [66].

**FIGURE 1:**
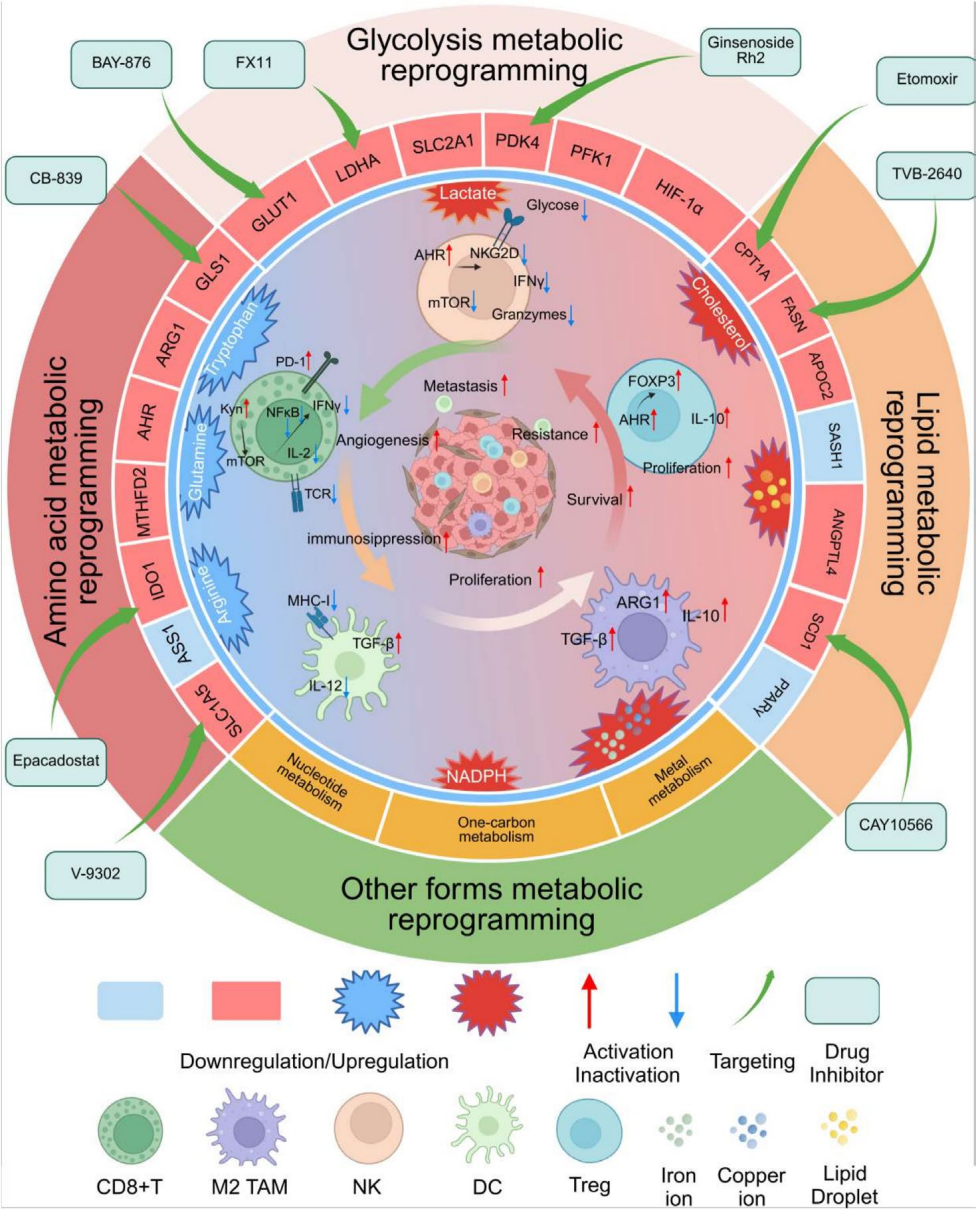
Crosstalk between metabolic remodeling and immune suppression in lung cancer: Potential targets. This schematic illustrates how reprogrammed metabolic pathways in lung cancer, including glycolysis, amino acid metabolism, lipid metabolism, nucleotide metabolism, one‐carbon metabolism, and metal metabolism, contribute to tumor progression and immune suppression. Dysregulation of key enzymes such as LDHA, GLS1, FASN, SLC1A5, MTHFD2, ASS1, and ACAT1 promotes immunosuppressive signaling through pathways like mTOR, AHR, PD‐1, and TGF‐β. These changes impair effector immune cells and support immunosuppressive cell populations. Representative metabolic inhibitors such as BAY‐876, CB‐839, and TVB‐2640 are shown as potential options for combined metabolic and immune therapies.

These trials underscore the growing recognition of the potential to integrate metabolic interventions with conventional therapies, aiming to improve clinical outcomes. However, further data are needed to establish the efficacy of these strategies across diverse patient populations and to refine the therapeutic approach for optimal clinical benefit. In Figure 1, we depict how metabolic reprogramming shapes the immune microenvironment in lung cancer and outline potential therapeutic strategies aimed at enhancing immunotherapy efficacy. Table 2 presents ongoing clinical trials investigating metabolic targets in lung cancer.

**TABLE 2:** Ongoing clinical trials investigating metabolic targets in lung cancer.

Drug/Combination	Types of metabolic targets	Targeted pathway/Mechanism	Phase	NCT number	Recruitment status	Efficacy data (OS/PFS/ORR, if available)
PX‐478	Glucose	HIF‐1α inhibitor	I	NCT01332686	Completed	NSCLC: tolerable, limited efficacy
Sapanisertib (TAK‐228)		mTORC1/2 inhibitor	I/II	NCT02052778	Completed	NSCLC: 1 PR, DCR 37%
Metformin + chemotherapy	Lipid	AMPK activator + chemotherapy	II	NCT02115464	Completed	NSCLC: no OS/PFS improvement
TVB‐2640 (Denifanstat)		FASN inhibitor (fatty acid synthesis)	II	NCT03808558	Recruiting	KRASmut NSCLC: DCR 60% at 12 weeks (interim)
Telaglenastat + Nivolumab	Amino acid	Glutaminase inhibitor + PD‐1 blockade	I/II	NCT02771626	Completed	NSCLC ORR 0%
Telaglenastat + Sapanisertib		Glutaminase inhibitor + mTORC1/2 inhibitor	I	NCT04250545	Recruiting (expansion)	DCR 62.5%, 1 PR (LUSC/NFE2L2 mutant)
CB‐839 + Cabozantinib		Glutaminase inhibitor + VEGFR/MET TKI	I	NCT02071862	Completed	Some SD in advanced NSCLC
Pegargiminase (ADI‐PEG20)		Arginine depletion (ASS1‐deficient tumors)	II	NCT01266018	Completed	SCLC ORR 0%, SD 18%
Pegargiminase + chemo		Arginine depletion + chemotherapy	I	NCT02029690	Completed	NSCLC ORR 47.6%, DCR 85.7%
Epacadostat + Pembrolizumab		IDO1 inhibitor + PD‐1 blockade	I/II	NCT03322540	Completed	NSCLC ORR 35% (small cohort)
Navoximod + Atezolizumab		IDO1 inhibitor + PD‐L1 blockade	I	NCT02471846	Completed	NSCLC ORR ~12%
BAY‐2402234	Others	DHODH inhibitor (pyrimidine metabolism)	I	NCT03404726	Recruiting	Early phase, data pending

## 6 NcRNAs in Metabolic Reprogramming and TIME Remodeling in Lung Cancer

Noncoding RNAs (ncRNAs), including long noncoding RNA (lncRNA), microRNA (miRNA), and circular RNA (circRNA), operate as multilayered regulators that couple metabolic rewiring to immune suppression in lung cancer, thereby shaping the TIME and fostering resistance to immunotherapy [71, 72].

**FIGURE 2:**
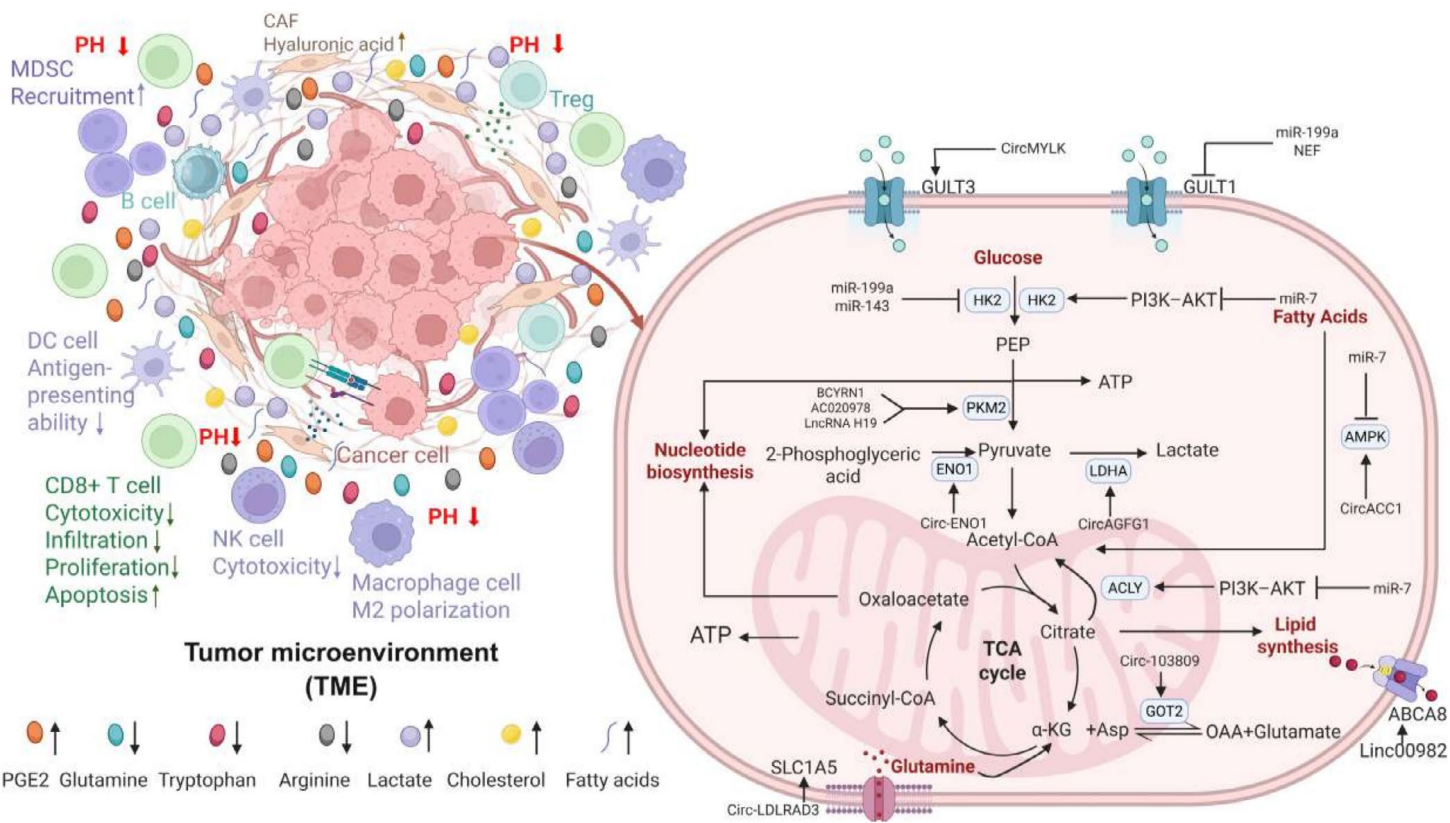
Noncoding RNAs–mediated regulation of metabolic–immune crosstalk in lung cancer. This schematic illustration summarizes the interplay between noncoding RNAs–mediated tumor metabolism and immune suppression in lung cancer. Enhanced glycolysis and lipid metabolic flux promote the accumulation of immunosuppressive metabolites such as lactate and PGE2. These metabolites impair the function of cytotoxic CD8^+^ T cells while supporting the expansion and activity of regulatory T cells (Tregs) and myeloid‐derived suppressor cells (MDSCs), thereby fostering an immunosuppressive tumor microenvironment. Noncoding RNAs are depicted as key regulators orchestrating both metabolic reprogramming and immune modulation.

Mechanistically, lncRNAs integrate metabolic and immune checkpoints: MALAT1 enhances glycolytic output via the mTOR‐4EBP1‐TCF7L2 axis and has been linked to immunosuppressive features in lung cancer, positioning metabolic flux upstream of myeloid and checkpoint modulation [73]. In parallel, NEAT1 epigenetically represses p53/cGAS/STING through DNMT1 to limit cytotoxic T‐cell infiltration in NSCLC, while NEAT1‐driven abnormal lipolysis via ATGL illustrates how ncRNA control of lipid metabolism intersects with immune evasion programs [74]. Beyond individual lncRNAs, lncRNAs elevate PD‐L1 by sponging PD‐L1‐targeting miRNAs and that PCAT1 can restrain cGAS/STING signaling via SOX2, directly connecting ncRNA networks to checkpoint dominance and impaired type‐I IFN responses [75]. Zhao et al. established the TIMELnc manual, which is a free and public manual for researchers to identify pivotal lncRNAs that are simultaneously correlated with tumor metabolism and immune cell infiltration based on a bioinformatic approach [76]. At the small‐RNA layer, miR‐21 confers a glycolytic advantage to NSCLC cells by suppressing FBP1, underscoring how miRNA tuning of rate‐limiting enzymes remodels nutrient competition and T‐cell function within the TIME. CircRNA‐centered ceRNA circuits reinforce these effects; in LUAD, circRNA‐002178 upregulates PD‐L1/PD‐1 signaling, directly augmenting immune checkpoint activity downstream of ncRNA metabolic‐immune coupling. Exosomal transfer further amplifies crosstalk: lncRNAs packaged in vesicles (e.g., UFC1 in NSCLC) stabilize oncogenic programs and disseminate metabolic‐immune cues across stromal and myeloid compartments. NcRNAs involved in metabolic reprogramming and immune resistance in lung cancer are listed in Table 3 [77, 78, 79, 80, 81, 82, 83, 84, 85, 86, 87, 88, 89, 90, 91]. In Figure 2, we depict ncRNAs–mediated regulation of metabolic–immune crosstalk in lung cancer.

**TABLE 3:** Noncoding RNA involved in metabolic reprogramming and immune resistance in lung cancer.

ncRNA	Target genes/Pathways	Metabolic function	Effects on immune response	References
Linc00982	Sponging miR‐183‐5p, upregulates ABCA8	Promote fatty acid metabolism	Friend	[77]
LncRNA H19	PKM2	Elevation of glucose metabolism	Foe	[78]
NEF	GLUT1	Repression of glucose metabolism	Friend	[79]
AC020978	Promotion of glycolysis by direct interaction with PKM2 and enhancing PKM2 protein stability Promotes the nuclear translocation of PKM2 and regulate PKM2‐enhanced HIF‐1α transcription activity	Elevation of glucose metabolism	Foe	[80]
miR‐143	Inhibiting HK2	Repression of the glucose metabolism	Friend	[81, 82]
MiR‐7	PI3K/AKT Signaling Pathway; MAPK Signaling Pathway; AMPK/LKB1 Signaling Pathway; NOVA2	Repression of the glucose metabolism	Friend	[83]
miR‐199a‐5p	Downregulating GLUT 1 and inhibiting HK2 and glycolysis	Repression of the glucose metabolism	Friend	[84]
CircACC1	Stabilize and promote the enzymatic activity of the AMPK holoenzyme by forming a ternary complex with the regulatory β and γ subunits	Modulate both fatty acid β‐oxidation and glycolysis	Foe	[85]
CircMYLK	Overexpression of GLUT3 by targeting miR‐195‐5p	Elevation of glucose metabolism	Foe	[86]
CircMAGI3	Overexpression of HDGF by targeting miR‐515‐5p	Elevation of glucose metabolism	Foe	[87]
CircAGFG1	Overexpression of HIF‐1α	Elevation of glucose metabolism	Foe	[88]
Circ‐ENO1	Regulating ENO1 expression by targeting miR‐22‐3p	Elevation of glucose metabolism	Foe	[89]
Circ‐LDLRAD3	Promoting glutamine metabolism through upregulation of SLC1A5 by targeting miR‐137	Elevation of amino acid metabolism	Foe	[90]
Circ‐103809	Overexpression of GOT2 by miR‐1298‐5p	Elevation of amino acid metabolism	Foe	[91]

Abbreviations: ABCA8, ATP binding cassette subfamily A member 8; AMPK, AMP‐activated protein kinase; ENO1, alpha‐enolase 1; GLUT1, glucose transporter type 1; GLUT3, glucose transporter type 3; GOT2, glutamic‐oxaloacetic transaminase 2; HDGF, heparin binding growth factor; HIF‐1α, hypoxia‐inducible factor 1‐alpha; HK2, Hexokinase 2; LKB1, liver kinase B1; MAPK, mitogen‐activated protein kinase; NOVA2, NOVA alternative splicing regulator 2; PI3K, phosphoinositide 3‐kinase; PKM2, pyruvate kinase M2; SLC1A5, solute carrier family 1 member 5.

Collectively, ncRNAs emerge as actionable hubs linking glycolysis and lipid metabolism to checkpoint regulation and innate/adaptive signaling, with clear biomarker and therapeutic implications for overcoming immune resistance.

## 7 Conclusion

Lung cancer remains a major therapeutic challenge due to its metabolic adaptability and immunosuppressive microenvironment. Tumor‐driven metabolic reprogramming disrupts antitumor immunity by altering key nutrient pathways involving glucose, lipids, AAs, and nucleotides, which contribute to immune evasion and limit the effectiveness of immunotherapy. Targeting these metabolic alterations represents a promising strategy to improve immunotherapy outcomes. Besides, ncRNAs act as hierarchical regulators that couple metabolic reprogramming to TIME remodeling, providing actionable biomarkers and therapeutic targets. However, monotherapies have shown limited efficacy, emphasizing the need for rational combination approaches. Future directions should also include identifying predictive biomarkers, characterizing subtype‐specific metabolic dependencies, integrating personalized metabolic profiling, and applying synthetic biology tools such as metabolically optimized CAR‐T and CAR‐NK cell therapies. A deeper understanding of the interaction between metabolism and immunity will facilitate the development of durable and personalized treatment strategies for lung cancer.

## Author Contributions

Yi Chen, Menglin Bai, and Ming Liu contributed equally to this work. Yi Chen, Menglin Bai, Ming Liu, and Leilei Wu drafted the manuscript and prepared the figures and tables. Ming Liu, Zhenshan Zhang, Chenxue Jiang, and Kejin Li revised the manuscript. Leilei Wu, Yaping Xu, and Yun Chen designed the project and provided resources to conduct the study. All authors have read and approved the final manuscript.

## Conflicts of Interest

The authors declare no conflicts of interest.

## Data Availability

The data that support the findings of this study are available from the corresponding author upon reasonable request.
